# How to Diminish the Numbers of Doctors

**Published:** 1893-09-30

**Authors:** F. Graham Bennett

**Affiliations:** 25, Savile Row


### EDITOR'S LETTER-BOX.

[Our correspondents are reminded that proxility is a great bar to publication, and that brevity of style and conciseness of statement greatly facilitate early insertion.]

### HOW TO DIMINISH THE NUMBERS OF DOCTORS.

Sir,—May I crave a little of your valuable space to give you my views on the subject of medical men. It is not so much the examination in arts as the overcrowding which exists owing to parents putting their sons, without counting the cost, into the medical professon. The popular idea is that as soon as the examinations are passed and the brass plate is bought, there is a living for a young man, but in reality unless he has private means to keep him until he has acquired the confidence of the public it simply means starvation. Even in country places, where the medical treatment used to be carried on by one medical practitioner, now there are dozens. It is no longer a case of the father handing down his profession to the son. The competition is so great that when these young men are admitted they find that if they cannot get a living by charging a high fee they are obliged to take a small one—and I do not blame them, as it is not nice to starve. What I do blame is the admitting indiscriminately into the profession a lot of people who practise massage. After a very short time these folks treat by themselves—without ever having seriously studied the subject or obtained a proper education—a number of diseases, wasting valuable time and money which might be better spent. Another grievance which might be remedied is the chemist giving advice in the minor ailments. I have been over and over again in a chemist's shop and seen him prescribe for almost everything. Then there are numerous patent medicines which patients have recourse to when they become dissatisfied with the treatment which they are deriving from their ordinary medical man. Besides all these circumstances there are a vastly greater number of medical men in the profession than there were 20 years ago. Last, not least, the hospitals are in open competition with the profession. They take small fees in the out-patients' room and admit patients as in-patients at £1 1s. a week. Country people having tedious complaints to deal with naturally think that if they can get board, treatment, &c., for £1 1s. a week they will not pay more. The hospitals should be either poor-law infirmaries or small proprietary hospitals, like French doctors have, where patients could be admitted at a small cost. If hospitals take funds from the public for free treatment they ought not to be allowed to charge. I think if all these matters were attended to we should not require a Defoe to reduce the medical profession.—I am, yours faithfully,

F. Graham Bennett, M.R.C.S., L.S.A.

25, Savile Row.

